# Compliance and barriers to the use of infection prevention and control measures among health care workers during COVID‐19 pandemic in Qatar: A national survey

**DOI:** 10.1111/jonm.13440

**Published:** 2021-09-14

**Authors:** Muna Abed Alah, Sami Abdeen, Nagah Selim, Dhouha Hamdani, Eman Radwan, Nahla Sharaf, Huda Al‐Katheeri, Iheb Bougmiza

**Affiliations:** ^1^ Community Medicine Department Hamad Medical Corporation (HMC) Doha Qatar; ^2^ Department of Family and Community Medicine Primary Health Care Corporation Doha Qatar; ^3^ Health Care Quality Management and Patient Safety Department Ministry of Public Health (MOPH) Doha Qatar; ^4^ Department of Strategic Planning and Performance Ministry of Public Health (MOPH) Doha Qatar; ^5^ Community Medicine Department Primary Health Care Corporation (PHCC) Doha Qatar; ^6^ Community Medicine Department, College of Medicine Sousse University Tunisia

**Keywords:** compliance, COVID‐19, hand hygiene, infection prevention and control, personal protective equipment

## Abstract

**Aim:**

To assess health care workers' compliance with infection prevention and control measures in different health care sectors in Qatar during COVID‐19 pandemic.

**Background:**

Being the first line of defence against COVID‐19 infection, health care workers are particularly at increased risk of getting infected. Compliance with infection prevention and control measures is essential for their safety and the safety of patients.

**Methods:**

A web‐based national survey was conducted between November 2020 and January 2021 targeting all health care workers in governmental, semi‐governmental and private health care sectors.

**Results:**

Of 1,757 health care workers, 49.9% were between 30 and 39 years of age; the majority (47.5%) were nurses. Participants reported a significant increase in the median self‐rated compliance scores during the pandemic compared with before it (*p* < .001). During the pandemic, 49.7% of health care workers were fully compliant with personal protective equipment (PPE) use; 83.1% were fully compliant with hand hygiene. Overall, 44.1% were fully compliant with infection prevention and control measures (PPE and hand hygiene). Nationality, health sector, profession and frequency of interactions with suspected or confirmed COVID‐19 cases were significantly associated with compliance with overall infection prevention and control measures. The most reported barriers were work overload and shortages of PPE and handwashing agents.

**Conclusions:**

Compliance of health care workers with infection prevention and control measures needs further improvement.

**Implications for Nursing Management:**

Frequent quality checks, provision of adequate supplies and behaviour change interventions are recommended strategies for hospital and nursing administrators to improve health care workers' compliance.

## 1 BACKGROUND

With the evolving spread of the coronavirus disease (COVID‐19), health care systems, resources and capacities worldwide become overwhelmed dealing with the rising numbers of infected persons. Health care workers—the first line of defence in the fight against COVID‐19—are particularly at risk of getting infected while taking care of infected patients (Gholami et al., 2021). A recent systematic review and meta‐analysis showed that the percentage of health care workers who tested positive for COVID‐19 among 28 studies was 51.7%, with a 15% rate of hospitalization and a 1.5% death rate (Gholami et al., 2021). In Qatar, the rates of COVID‐19 infection and hospitalization among health care workers are 10.6% and 11.6%, respectively (Alajmi et al., 2020). Standard precautions such as proper use of personal protective equipment (PPE), proper hand hygiene and respiratory hygiene practices are essential preventive measures against the spread of the infection in health care facilities. The large number of COVID‐19 infected cases among health care workers was attributed to inadequate personal protection of health care workers at the beginning of the pandemic, shortage of PPE, and inadequate training of health care workers on the appropriate infection prevention and control measures (Shanghai International Forum for Infection Control and Prevention, 2020).

Low compliance with infection prevention and control measures may have negative consequences for workers, patients and institutions such as the occurrence of occupational accidents, health care‐associated infections and institutional damage (Askarian et al., 2004; I. Jeong et al., 2008; Oliveira et al., 2009; World Health Organization [WHO], 2011). Health care‐associated infections can result in prolonged hospital stays, long‐term disability, massive additional costs for health systems and organizations, and unnecessary deaths (WHO, 2011). Compliance with PPE among health care workers during COVID‐19 pandemic varied among different studies, ranging from 54% to over 95% (Ashinyo et al., 2021; Darwish et al., 2021; Michel‐Kabamba et al., 2020; Neuwirth et al., 2020).

According to current evidence, the SARS‐CoV 2 virus is transmitted between people through respiratory droplets and contact routes. Transmission can occur by direct contact with infected people and indirect contact with surfaces in the immediate environment (Chan et al., 2020; Huang et al., 2020; Li et al., 2020; Liu et al., 2020). The World Health Organization (WHO) recommended droplet and contact precautions (including the use of a medical mask, eye protection (goggles) or facial protection (face shield), a clean, non‐sterile, long‐sleeved gown and gloves) for health care workers caring for suspected or confirmed COVID‐19 patients, and airborne precautions using N95 respirator or equivalent in addition to contact precautions for settings in which aerosol generating procedures (AGPs) are performed. It also emphasized the importance of practicing hand hygiene (WHO, 2020a). Qatar formulated national infection prevention and control guidelines for COVID‐19 in accordance with the WHO and Centers for Disease Control's (CDC) recommendations.

To the best of our knowledge, studies assessing compliance with the proper use of infection prevention and control measures among health care workers during this pandemic are limited, particularly in the Middle East. This is the first national study in Qatar to address this issue. It is expected that compliance with the use of PPE and hand hygiene practices changes after an epidemic, as this was evident from previous infectious outbreaks when significant improvements in compliance were noted (G. Jeong et al., 2016; Wong & Tam, 2005). So, addressing the changes in compliance during the current pandemic is worth investigating. We aimed to assess health care workers' compliance with the proper use of PPE and hand hygiene practices in different health care sectors in Qatar (governmental, semi‐governmental and private sectors) during COVID‐19 pandemic and explore the barriers to the proper use of such measures.

## 2 METHODS

### 2.1 Study design, setting and the target population

A national web‐based cross‐sectional study was conducted between November 2020 and January 2021. The target population included health care workers at governmental, semi‐governmental and private health care sectors. In Qatar, health care services are provided by these three sectors. The governmental sector provides primary health care services at the level of Primary Health Care Corporation (PHCC) through 27 health centres distributed all over the country, and secondary and tertiary care through Hamad Medical Corporation with a number of designated hospitals. The semi‐governmental sector includes six health care facilities. The private sector includes over 40 private hospitals and clinics. We included health care workers in clinical positions (physicians, nurses, dentists, pharmacists and allied health professionals), while excluded those in administrative positions.

### 2.2 Study procedure

A web‐based self‐administered survey was developed using Microsoft Forms software. Because of the low response rate generally encountered in web‐based surveys and in order to improve the external validity of our study, we invited all eligible health care workers in PHCC (representing a major part of the governmental sector), semi‐governmental and private facilities to take the survey. They were contacted via e‐mail with an information letter and a link to the electronic version of the questionnaire. The letter stated the purpose of the study, and that the participation is voluntary. Taking the survey implied informed consent, and participants were free to terminate the survey at any time they desired. The survey was anonymous, and confidentiality of information was assured. Weekly reminders were sent to maximize the response rate.

### 2.3 Study questionnaire

We developed a questionnaire that was adopted from different surveys (Chia et al., 2005; Majeed, 2018; Schwartz et al., 2014; Shimokura et al., 2006; WHO, 2020c) in English. Face and content validities were assured by experts in the field. It consisted of three sections. The first one addressed the socio‐demographic data for the participants (age, gender, nationality, profession, clinical experience and health care facility), in addition to general COVID‐19‐related information such as having a friend or a relative infected with COVID‐19, the status of training on proper use of PPE and hand hygiene practices, and the frequency of dealing with suspected or confirmed COVID‐19 cases. The second and third sections assessed health care workers' compliance with the proper use of infection prevention and control measures (PPE and hand hygiene) using a checklist adopted from WHO risk assessment tool for health care workers in the context of COVID‐19 (WHO, 2020c), and the barriers to the proper use, respectively.

### 2.4 Study variables

To assess the compliance of health care workers with infection prevention and control measures, they were asked about the frequency of using each PPE item when dealing with suspected or confirmed cases or while performing an AGP for a suspected or confirmed case using a five‐point‐Likert scale (always as recommended, often, sometimes, seldom, never). And were asked about the frequency of performing hand hygiene (using similar Likert scale) at five moments that are as follows: before touching a patient, before any clean or aseptic procedure is performed, after exposure to body fluid, after touching a patient and after touching patient's surroundings. Health care workers who answered all the questions as ‘always as recommended’ were considered as fully compliant. We also asked the participants to rate their overall perceived compliance with proper use of PPE and hand hygiene before and during the pandemic on a ten‐point scale (from 0 to 9, on which 0 indicates no compliance, and 9 indicates full compliance). Barriers to the appropriate use of infection prevention and control measures as recommended were assessed by asking health care workers to select one or more options from a list of barriers for PPE and hand hygiene separately. They were also able to specify other barriers that were not listed.

### 2.5 STATISTICAL ANALYSIS

Data analysis was performed using IBM SPSS Statistics for Windows, Version 26.0. Armonk, NY: IBM Corp. Descriptive statistics were presented as frequencies and percentages for categorical variables. Continuous not normally distributed variables were presented as medians and interquartile ranges. Chi‐square test was used to determine the differences between categorical variables. The Wilcoxon signed rank test was used to test the differences in the self‐rated compliance with infection prevention and control measures before and during COVID‐19 pandemic taking into consideration the self‐rated compliance on the ten‐point scale as an ordinal dependent variable. Rank biserial correlation was calculated to measure the effect size for these comparisons (small 0.10 to <0.30, medium 0.30 to <0.50, large ≥0.50). Three multivariable logistic regression models were executed to determine the predictors of full compliance with infection prevention and control measures, one for appropriate use of PPE, one for hand hygiene, and one for overall infection prevention and control measures (both PPE and hand hygiene). The associations between risk factors and outcomes were presented as adjusted odds ratios (ORs) and 95% confidence intervals (95% CIs). Goodness of fit was assessed using Hosmer–Lemeshow test. *p* values less than .05 were considered significant.

### 2.6 Ethical approval

This study was performed in line with the principals of Declaration of Helsinki. Approval was obtained from the relevant health institutions under protocol ID PHCC/DCR/2020/07/073.

## 3 RESULTS

### 3.1 Socio‐demographic characteristics and general information

As shown in Table 1, the survey was completed by 1,757 health care workers: of them, 757 (43.1%) from the governmental sector (PHCC), 480 (27.3%) from the semi‐governmental sector, and 520 (29.6%) from the private sector. Majority (49.9%) were between 30 and 39 years of age; 1,192 (67.8%) were females. Over 60 nationalities were reported, with the top three being Filipino (29.8%), Indian (27.4%) and Egyptian (6.4%). Only 32 (1.8%) Qatari health care workers participated in this study. Nurses accounted for the majority of health care workers (47.5%), followed by allied health professionals (22%) and physicians (20.1%). Of all participants, 1,573 (89.5%) reported five years or more of clinical experience. 91.3% and 95.6% of health care workers admitted receiving a training on proper PPE use and proper hand hygiene practices in the preceding year, respectively. Over one third (35.2%) of health care workers reported frequent interactions with suspected or confirmed COVID‐19 cases (every work shift or most of their work shifts). And about three quarters (77.1%) were aware of a relative, friend or colleague diagnosed with COVID‐19.

**TABLE 1:** Socio‐demographic profiles and background information of the participants by health care sector

Variable		Health sector			Totals*N* = 1757No (%)	*χ* ^2^ test *p* value
		Governmental*n* = 757No (%)	Private*n* = 520No (%)	Semi‐governmental*n* = 480No (%)		
Age categories	<30	66 (8.7)	77 (14.8)	45 (9.4)	188 (10.7)	<.001
	30–39	380 (50.2)	307 (59.0)	189 (39.4)	876 (49.9)	
	40–49	212 (28.0)	98 (18.8)	147 (30.6)	457 (26.0)	
	≥50	99 (13.1)	38 (7.3)	99 (20.6)	236 (13.4)	
Gender	Female	475 (62.7)	376 (72.3)	341 (71.0)	1,192 (67.8)	<.001
	Male	282 (37.3)	144 (27.7)	139 (29.0)	565 (32.2)	
Nationality^a^ (by regional classification)	Asia‐Pacific	417 (55.1)	436 (83.8)	207 (43.1)	1,060 (60.3)	<.001
	Americas	6 (0.8)	6 (1.2)	51 (10.6)	63 (3.6)	
	Europe	36 (4.8)	37 (7.1)	132 (27.5)	205 (11.7)	
	Middle East–North Africa	240 (31.7)	38 (7.3)	62 (12.9)	340 (19.4)	
	Sub‐Saharan Africa	58 (7.7)	3 (0.6)	28 (5.8)	89 (5.1)	
Profession	Allied health professional	213 (28.1)	64 (12.3)	109 (22.7)	386 (22.0)	<.001
	Dentist	30 (4.0)	19 (3.7)	1 (0.2)	50 (2.8)	
	Nurse	267 (35.3)	315 (60.6)	252 (52.5)	834 (47.5)	
	Pharmacist	97 (12.8)	20 (3.8)	17 (3.5)	134 (7.6)	
	Physician	150 (19.8)	102 (19.6)	101 (21.0)	353 (20.1)	
Clinical experience	Less than 1 year	20 (2.6)	9 (1.7)	1 (0.2)	30 (1.7)	<.001
	1–4 years	67 (8.9)	67 (12.9)	20 (4.2)	154 (8.8)	
	5 or more years	670 (88.5)	444 (85.4)	459 (95.6)	1,573 (89.5)	
Frequency of interaction with COVID‐19 suspected or confirmed patients	Never	86 (11.4)	136 (26.2)	170 (35.4)	392 (22.3)	<.001
	Some of the shifts	226 (29.9)	276 (53.1)	245 (54.0)	747 (42.5)	
	Most of the shifts	99 (13.1)	60 (11.5)	30 (6.3)	189 (10.8)	
	Every shift	346 (45.7)	48 (9.2)	35 (7.3)	429 (24.4)	
Training on proper PPE use in the past year	No	89 (11.8)	51 (9.8)	13 (2.7)	153 (8.7)	<.001
	Yes	668 (88.2)	469 (90.2)	467 (97.3)	1,604 (91.3)	
Training on proper hand hygiene practices in the past year	No	47 (6.2)	24 (4.6)	6 (1.3)	77 (4.4)	<.001
	Yes	710 (93.8)	496 (95.4)	474 (98.8)	1,680 (95.6)	

Abbreviation: *χ* ^2^, chi square; PPE, personal protective equipment.

^a^ More than 60 different nationalities were reported.

### 3.2 Compliance with infection prevention and control measures

When participants were asked to rate their compliance with infection prevention and control measures on a ten‐point scale, before and during the pandemic, there was a significant increase in the median self‐rated compliance scores during the pandemic compared with before it (median score: 7 before and 9 during, for PPE), and (median score: 8 before and 9 during, for hand hygiene), with *p* values < .001 and large effect sizes (*r* = .87 and .89), respectively. We assessed compliance with infection prevention and control measures using a checklist adopted from WHO risk assessment tool for health care workers in the context of COVID‐19 (WHO, 2020c). According to this checklist, 52.6% (95% CI: 49.9–55.4) were fully compliant with PPE use during patient interactions with suspected or confirmed COVID‐19 cases, 73.2% (95% CI: 70.4–76.0) while performing an AGP for a suspected or confirmed COVID‐19 case, and 49.7% (95% CI 46.5–52.8) during both patient interactions and while performing an AGP. Regarding compliance with hand hygiene, 83.1% (95% CI 81.3–84.8) of health care workers were fully compliant with hand hygiene during the five moments. Overall, 44.1% (95% CI: 41.0–47.2) were fully compliant with infection prevention and control measures (with both PPE and hand hygiene).

### 3.3 Predictors of compliance with infection prevention and control measures

Three multivariable logistic regression models were executed to determine the predictors of compliance with infection prevention and control measures. One for compliance with PPE (during both interactions with suspected or confirmed COVID‐19 cases and while performing an AGP), one for compliance with hand hygiene at the five moments, and a third one for compliance with overall infection prevention and control measures (with both PPE and hand hygiene). All models were of good fit and were statistically significant (*p* values < .001) compared to the null model. The selection of independent variables to be included in the models was based on clinical and statistical relevance. In the first model (Table 2), nationality, health sector, profession and frequency of interactions with suspected or confirmed COVID‐19 cases were found to be significantly and independently associated with compliance with PPE. The following were less likely to be fully compliant: nationalities of Middle Eastern–North African origin compared to those of Asia‐Pacific origin (OR 0.44, 95% CI 0.30–0.65, *p* < .001), pharmacists compared to physicians (OR 0.16, 95% CI 0.07–0.38, *p* < .001), and health care workers at the private sector compared to those at the governmental sector (PHCC). On the other hand, dentists were more likely to be fully compliant with PPE compared to physicians (OR 6.23, 95% CI 2.37–16.38, *p* < .001), so as health care workers who deal with suspected or confirmed COVID‐19 cases frequently (every shift) compared to those who never deal with such cases (OR 1.99, 95% CI 1.18–3.36, *p* = .010). In the second model (Table 3), compliance with hand hygiene was significantly associated with gender, nationality, profession and previous training on hand hygiene. Males were less likely to be compliant than females (adjusted OR 0.71, 95% CI: 0.52–0.95, *p* = .022), as well as those with nationalities of all origins compared to those with nationalities of Asia‐Pacific origin. On the other hand, allied health professionals were more likely to be compliant with hand hygiene compared to physicians (adjusted OR 2.06, 95% CI: 1.32–3.20, *p* = .001), and health care workers who received previous training on hand hygiene were more than two times more likely to be compliant compared to those who did not (adjusted OR 2.42, 95% CI: 1.44–4.07, *p* = .001). In the third model (Table 4), the overall compliance with infection prevention and control measures (both PPE and hand hygiene) was also significantly associated with nationality, profession, health sector and frequency of interactions with suspected or confirmed COVID‐19 cases. Health care workers of Middle Eastern, North African and Sub‐Saharan African origins were less likely to be compliant compared to those of Asia‐Pacific origin. Dentists were about six times more likely to be compliant compared to physicians (adjusted OR 5.84, 95% CI: 2.44–14.00, *p* < .001). Compared to health care workers in the governmental sector, those working in the semi‐governmental sector were more likely to be fully compliant with infection prevention and control measures (adjusted OR 1.63, 95% CI: 1.09–2.43, *p =* .026), whereas those in the private sector were less likely to be fully compliant (adjusted OR 0.63, 95% CI: 0.43–0.92, *p* = .017). Those dealing frequently (every shift) with suspected or confirmed COVID‐19 cases were about two times more likely to be fully compliant than those who never deal with such cases (adjusted OR 2.02, 95% CI: 1.19–3.42, *p* = .009).

**TABLE 2:** Determinants and predictors of full compliance with PPE using chi‐square test and multiple logistic regression analysis

Variable		PPE compliance^a^			
		Fully compliantNo (%)^b^	*χ* ^2^ test *p* value	Multivariable regression analysis	
				AOR (95% CI)	*p* value
Age categories	<30	39 (39.0)	.280	1 (reference)	
	30–39	256 (49.0)		1.13 (0.69–1.85)	.641
	40–49	136 (50.6)		1.18 (0.67–2.07)	.565
	≥50	70 (59.3)		1.66 (0.87–3.17)	.125
Gender	Female	336 (50.2)	.611	1 (reference)	
	Male	165 (48.5)		0.98 (0.71–1.34)	.879
Nationality (by regional classification)	Asia‐Pacific	323 (52.7)	<.001	1 (reference)	
	Americas	22 (66.7)		1.30 (0.57–3.03	.546
	Europe	69 (56.1)		0.80 (0.49–1.32)	.389
	Middle East‐North Africa	71 (36.2)		0.44 (0.30–0.65)	<.001
	Sub‐Saharan Africa	16 (36.4)		0.54 (0.26–1.13)	.101
Profession	Allied health professional	81 (43.8)	<.001	0.72 (0.45–1.16)	.175
	Dentist	28 (82.4)		6.23 (2.37–16.38)	<.001
	Nurse	276 (53.5)		1.00 (0.65–1.54)	.99
	Pharmacist	7 (11.9)		0.16 (0.07–0.38)	<.001
	Physician	109 (50.7)		1 (reference)	
Health sector	Governmental	257 (51.1)	<.001	1 (reference)	
	Private	93 (37.1)		0.50 (0.34–0.72)	<.001
	Semi‐governmental	151 (59.2)		1.41 (0.94–2.10)	.094
Clinical experience	Less than 1 year	11 (52.4)	.076	1 [reference]	
	1–4 years	30 (37.5)		0.84 (0.29–2.43)	.747
	5 or more years	460 (50.7)		1.01 (0.39–2.62)	.987
Relative or friend diagnosed with COVID‐19	No	110 (51.6)	.513	1 (reference)	
	Yes	391 (49.1)		0.90 (0.65–1.26)	.541
Frequency of interaction with COVID‐19 suspected or confirmed patients	Never	48 (41.7)	.007	1 (reference)	
	Some of the shifts	208 (47.8)		1.33 (0.84–2.11)	.227
	Most of the shifts	58 (44.3)		1.33 (0.75–2.36)	.323
	Every shift	187 (57.0)		1.99 (1.18–3.36)	.010
Training on proper PPE use in the past year	No	27 (36.5)	.019	1 (reference)	
	Yes	474 (50.7)		1.08 (0.61–1.90)	.797

Abbreviations: AOR, adjusted odds ratio; CI, confidence interval; PPE, personal protective equipment; *χ* ^2^, chi square.

^a^ The outcome of the regression model is overall compliance with PPE (*N* = 1,009) including compliance during interaction with suspected or confirmed COVID‐19 cases and while performing an aerosol generating procedure for suspected or confirmed COVID‐19 case.

^b^ These are row percentages (fully compliant/[fully compliant + not fully compliant]) for each variable subcategories.

**TABLE 3:** Determinants and predictors of full compliance with hygiene using chi‐square test and multiple logistic regression analysis

Variable		Hand hygiene compliance^a^			
		Fully compliantNo (%)^b^	*χ* ^2^ test *p* value	Multivariable regression analysis	
				AOR (95% CI)	*p* value
Age categories	<30	155 (82.4)	.35	1 (reference)	
	30–39	749 (85.5)		1.56 (0.71–1.88)	.562
	40–49	371 (81.2)		1.34 (0.78–2.31)	.293
	≥50	185 (78.4)		1.30 (0.70–2.39)	.405
Gender	Female	1,020 (85.6)	<.001	1 (reference)	
	Male	440 (77.9)		0.71 (0.52–0.95)	.022
Nationality (by regional classification)	Asia‐Pacific	950 (89.6)	<.001	1 (reference)	
	Americas	49 (77.8)		0.39 (0.20–0.79)	.009
	Europe	158 (77.1)		0.45 (0.28–0.71)	.001
	Middle East‐North Africa	244 (71.8)		0.38 (0.27–0.55)	<.001
	Sub‐Saharan Africa	59 (66.3)		0.26 (0.15–0.44)	<.001
Profession	Allied health professional	338 (87.6)	<.001	2.06 (1.32–3.20)	.001
	Dentist	42 (84.0)		1.70 (0.74–3.93)	.212
	Nurse	727 (87.2)		1.45 (0.98–2.16)	.064
	Pharmacist	96 (71.6)		1.28 (0.77–2.11)	.345
	Physician	257 (72.8)		1 (reference)	
Health sector	Governmental	606 (80.1)	.005	1 (reference)	
	Private	452 (86.9)		1.25 (0.86–1.84)	.246
	Semi‐governmental	402 (83.8)		1.28 (0.87–1.89)	.212
Clinical experience	Less than 1 year	26 (86.7)	.263	1 (reference)	
	1–4 years	121 (78.6)		0.73 (0.23–2.34)	.600
	5 or more years	1,313 (83.5)		1.07 (0.35–3.22)	.910
Relative or friend diagnosed with COVID‐19	No	344 (85.4)	.167	1 (reference)	
	Yes	1,116 (82.4)		0.83 (0.60–1.15)	.258
Frequency of interaction with COVID‐19 suspected or confirmed patients	Never	331 (84.4)	.658	1 (reference)	
	Some of the shifts	615 (82.3)		0.84 (0.59–1.20)	.348
	Most of the shifts	161 (85.2)		1.12 (0.66–1.89)	.673
	Every shift	353 (82.3)		1.02 (0.56–1.59)	.921
Training on proper hand hygiene practices in the past year	No	48 (62.3)	<.001	1 (reference)	
	Yes	1,412 (84.0)		2.42 (1.44–4.07)	.001

Abbreviations: AOR, adjusted odds ratio; CI, confidence interval; *χ* ^2^, chi square.

^a^ The outcome of the regression model is the compliance with hand hygiene (*N* = 1757).

^b^ These are row percentages (fully compliant/[fully compliant + not fully compliant]) for each variable subcategories.

**TABLE 4:** Determinants and predictors of full compliance with overall IPC measures using chi‐square test and multiple logistic regression analysis

Variable		Overall IPC compliance^a^			
		Fully compliantNo (%)^b^	*χ* ^2^ test *p* value	Multivariable regression analysis	
				AOR (95% CI)	*p* value
Age categories	<30	34 (34.0)	.130	1 (reference)	
	30–39	230 (44.1)		1.18 (0.71–1.95)	.522
	40–49	123 (45.7)		1.51 (0.86–2.67)	.155
	≥50	58 (49.2)		1.80 (0.94–3.45)	.076
Gender	Female	298 (44.5)	.692	1 (reference)	
	Male	147 (43.2)		1.11 (0.81–1.54)	.519
Nationality (by regional classification)	Asia‐Pacific	304 (49.6)	<.001	1 (reference)	
	Americas	15 (45.5)		0.57 (0.26–1.27)	.167
	Europe	58 (47.2)		0.64 (0.39–1.06)	.081
	Middle East‐North Africa	55 (28.1)		0.35 (0.24–0.53)	<.001
	Sub‐Saharan Africa	13 (29.5)		0.43 (0.20–0.90)	.026
Profession	Allied health professional	76 (41.1)	<.001	1.00 (0.61–1.62)	.989
	Dentist	25 (73.5)		5.84 (2.44–14.00)	<.001
	Nurse	252 (48.8)		1.27 (0.82–2.00)	.284
	Pharmacist	7 (11.9)		0.28 (0.12–0.82)	.005
	Physician	85 (39.5)		1 (reference)	
Health sector	Governmental	225 (44.7)	<.001	1 (reference)	
	Private	87 (34.7)		0.63 (0.43–0.92)	.017
	Semi‐governmental	133 (52.2)		1.63 (1.09–2.43)	.026
Clinical experience	Less than 1 year	11 (52.4)	.074	1 (reference)	
	1–4 years	26 (32.5)		0.65 (0.22–1.88)	.421
	5 or more years	408 (44.9)		0.78 (0.30–2.04)	.619
Relative or friend diagnosed with COVID‐19	No	98 (46.0)	.528	1 (reference)	
	Yes	347 (43.3)		0.93 (0.67–1.29)	.666
Frequency of interaction with COVID‐19 suspected or confirmed patients	Never	44 (38.3)	.005	1 (reference)	
	Some of the shifts	177 (40.7)		1.16 (0.73–1.84)	.537
	Most of the shifts	53 (40.5)		1.36 (0.77–2.41)	.295
	Every shift	171 (52.1)		2.02 (1.19–3.42)	.009
Training on proper PPE use in the past year	No	22 (29.7)	.010	1 (reference)	
	Yes	423 (45.2)		1.08 (0.57–2.06)	.811
Training on proper hand hygiene practices in the past year	No	13 (27.7)	.020	1 (reference)	
	Yes	432 (44.9)		1.29 (0.59–2.82)	.520

Abbreviations: AOR, adjusted odds ratio; CI, confidence interval; IPC, infection prevention and control; PPE, personal protective equipment; *χ* ^2^, chi square.

^a^ The outcome of the regression model is the overall compliance with IPC measures (both PPE and hand hygiene) (*N* = 1,009).

^b^ These are row percentages (fully compliant/[fully compliant + not fully compliant]) for each variable subcategories.

### 3.4 Barriers to proper use of infection prevention and control measures

**FIGURE 1:**
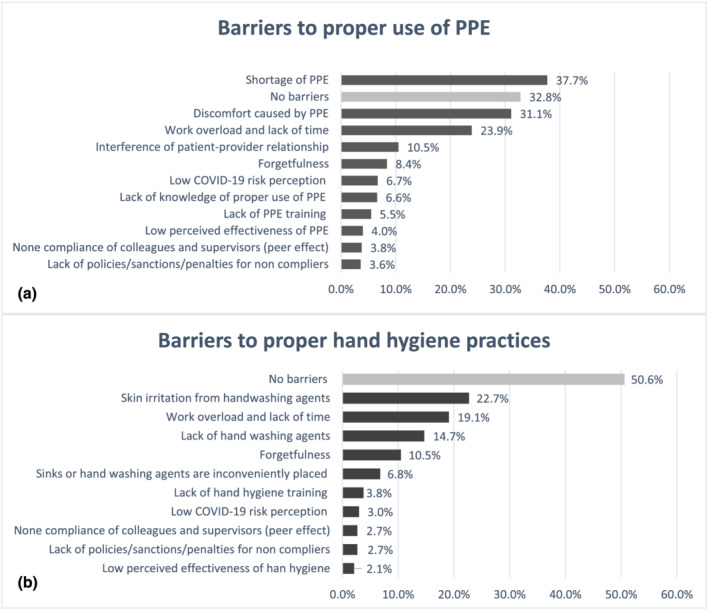
Barriers to infection prevention and control measures ((a) to proper personal protective equipment [PPE] use and (b) to proper hand hygiene practices)

Regarding the barriers to the proper use of PPE, the most reported barriers were shortage of PPE (37.7%), discomfort caused by PPE such as N95 respirators or face shields (31.3%), and work overload and lack of time (23.9%). For hand hygiene, skin irritation caused by handwashing agents was the most reported barrier (22.7%), followed by work overload and lack of time (19.1%), and shortage of handwashing agents (14.7%). On the other hand, 32.8% and 50.6% of participants reported no barriers at all for PPE use or practicing hand hygiene, respectively (Figure 1). Higher proportions of health care workers reported shortages of PPE and handwashing agents as barriers in the governmental sector compared to the other sectors. Proportions of health care workers who reported shortage of PPE were 42.3%, 33.7% and 35% for governmental, private and semi‐governmental sectors, respectively, whereas 18.4%, 12.3% and 11.5% reported shortage of handwashing agents.

## 4 DISCUSSION

Being the first line of defence against COVID‐19 infection, health care workers are particularly at increased risk of getting infected. Compliance with infection prevention and control measures is critically essential for their safety and the safety of their patients. In this study, we assessed health care workers' compliance in different health care sectors with proper use of PPE and hand hygiene practices as reported by them. The majority of health care workers in this study were nurses, with nurse: physician ratio of 2.4:1 reflecting almost the same ratio in the health care workers' population in Qatar, which is about 2.8:1. We found a significant improvement in health care workers' perceived compliance with infection prevention and control measures since the start of the pandemic. This might have resulted from the heightened awareness of the importance of complying with PPE and hand hygiene during COVID‐19 at international and national levels, and from the greater perceived threat health care workers are experiencing during this emerging serious infection. This finding matches what was reported in a study in China (Lai et al., 2020). Comparing our results with those of a recently published study conducted in Ghana that utilized the same assessment tool (WHO checklist), the compliance of health care workers with PPE in our study was found to be lower during both patient interactions (52.6%) and while performing an AGP (73.2%) compared with 90.6% and 97.5% in the other study respectively (Ashinyo et al., 2021), whereas similar compliance rates were found with hand hygiene practices (Ashinyo et al., 2021). On the other hand, the compliance with PPE during patient interactions in our study was similar to what was reported in another study in the Democratic Republic of the Congo (Michel‐Kabamba et al., 2020) and was much higher while performing AGPs compared to another study in the United States (Darwish et al., 2021). In this study, pharmacists were found less likely to be fully compliant with proper use of PPE than physicians, which is consistent with the results of Ghana study (Ashinyo et al., 2021). One explanation might be that pharmacists are less likely to have direct contact with patients in general and with suspected or confirmed COVID‐19 cases. In addition, the duration of their contact is usually short and in most cases like in hospitals, patient's family or friends are the ones who attend the pharmacy for medication pick up upon discharge not the patient him/herself. Also, at almost all pharmacies in Qatar, most of the contacts happen through glass shields that might be perceived as protective by many pharmacists against countering infection. On the other hand, dentists were found more likely to be compliant with PPE and with overall infection prevention and control measures than physicians. This might be explained by the closer contact dentists have with their patients while managing them, as their job involves more contact with aerosols and droplets produced during many dental procedures that have the potential to spread the infection to dental personnel. This will force dentists to be more fully compliant with infection prevention and control measures in a step to protect themselves from getting infected. Health care workers in the governmental sector showed higher compliance rates with overall infection prevention and control measures and with PPE than those in the private sector. This can be explained by the fact that health care workers in the governmental sector in Qatar deal more frequently with suspected or confirmed COVID‐19 cases than other sectors as shown in Table 1. In Qatar, COVID‐19‐positive cases are managed in the governmental sector. Private health care facilities deal with suspected cases, but such cases are transferred to the governmental sector once confirmed. This finding is also supported by another finding in our study that showed that those who deal more frequently with suspected or confirmed cases were more likely to be compliant, which is also consistent with established findings in the literature (Brooks et al., 2020). We found that lack of time, discomfort caused by certain types of PPE, shortage of PPE, and skin irritation caused by handwashing agents as barriers for health care workers' compliance with infection prevention and control measures similar to the existing literature (Ahmed et al., 2020; Ataiyero et al., 2019; Fan et al., 2020; Houghton et al., 2020; WHO, 2009). We found higher proportions of health care workers reporting shortages of PPE and handwashing agents in the governmental sector compared with other sectors. This finding is expected in the light of global shortages of PPE and the greater burden of patients at the governmental sector where all positive COVID‐19 cases are managed compared to other sectors. WHO has warned that shortages of PPE caused by increasing demand, panic buying, and misuse is putting the lives of health care workers at risk from the current COVID‐19 pandemic and other infectious diseases. Assuring appropriate usage of PPE by health care workers and avoiding overuse are critically important. For this, the WHO issued guidance for rational use of PPE in health care settings and the effective management of supply chains (WHO, 2020b). Despite the emergence of COVID‐19 vaccines, compliance with proper infection prevention and control measures by all health care workers is of paramount importance, as these vaccines are still surrounded by uncertainties and under continuous investigation.

### 4.1 Strengths and limitations

This study had several strengths. First, it is the first national study in Qatar, and one of the few studies worldwide to address this important issue during the current COVID‐19 pandemic. Second, we were able to recruit an acceptable number of health care workers from all health care sectors strengthening our confidence in generalizing our results to the health care workers population in Qatar. Although this study provides new insights on the use of infection prevention and control measures by health care workers during this emerging challenging pandemic of COVID‐19, we do acknowledge some limitations. First, the data were collected by self‐reporting by health care workers not by direct observation of their practices, which might lead to recall, and social‐desirability bias. So, the detected compliance rates should be viewed cautiously. However, online surveys were the only and safest means to collect data for research purposes in light of national recommendations of keeping physical distancing as a way to contain the spread of the infection. Second, with the cross‐sectional design of this study, we could not establish how compliance with infection prevention and control measures translates into lower incidence of COVID‐19 infection. Lastly, individual institutional infection prevention and control recommendations and instructions might influenced health care workers' compliance and affected our results.

## 5 CONCLUSION

Despite the significant improvement in the perceived self‐rated compliance of health care workers with different infection prevention and control measures (PPE and hand hygiene), their compliance with overall infection prevention and control measures was found to be moderate (44.1%). The highest compliance rate was found with hand hygiene at the five moments (83.1%), and the lowest with PPE during patient interactions (52.6%). This study shows gaps in infection prevention and control compliance across different health professional groups with higher compliance rates among dentists and lower compliance with pharmacists compared to physicians. Health care sector, nationality and frequency of dealing with suspected or confirmed COVID‐19 cases were found to be predictors of compliance with PPE and with overall infection prevention and control measures. On the other hand, gender, nationality, profession and previous training on hand hygiene were found to be associated with hand hygiene compliance. Several barriers were reported to the proper use of infection prevention and control measures including work overload and shortages of PPE and handwashing agents. Compliance of health care workers with infection prevention and control measures needs to be further improved.

### 5.1 Implications for nursing management

Frequent quality checks, continuous monitoring, provision of adequate supplies (PPE, and handwashing agents) and behaviour change interventions are top strategies that can be enforced by policymakers, safety managers of health care institutions, hospital and nursing administrators to improve compliance. Conducting further research that involves direct observation of infection prevention and control related practices is needed.

## CONFLICT OF INTEREST

None.

## FUNDING INFORMATION

This research received no specific grant from any funding agency in the public, commercial, or not‐for‐profit sectors.

## ETHICAL APPROVAL

This study was performed in line with the principals of Declaration of Helsinki. Approval was obtained from the Institutional Review Board of the Primary Health Care Corporation (PHCC) under protocol ID PHCC/DCR/2020/07/073 to carry out the survey at PHCC level, and an exempt certificate was obtained from The Health Research Governance Department at Ministry of Public Health (MOPH) to carry out the survey at the semi‐governmental and private sectors.

## Data Availability

The data that support the findings of this study are available from the corresponding author upon reasonable request.
